# A case report of radiofrequency ablation of fascicular ventricular tachycardia after viral myocarditis

**DOI:** 10.1097/MD.0000000000042606

**Published:** 2025-05-23

**Authors:** Wei Liu, Yu Bing Wang, Jie Feng, Yun Chen

**Affiliations:** a Department of Cardiovascular Medicine, Affiliated Hospital of North Sichuan Medical College, Nanchong, Sichuan, China.

**Keywords:** fascicular ventricular tachycardia, radiofrequency ablation, vital myocarditis

## Abstract

**Rationale:**

Viral myocarditis is more common in clinical practice with arrhythmia, but it is rare to have idiopathic ventricular tachycardia. Literature review shows that there are no reports on the treatment of idiopathic ventricular tachycardia complicated by viral myocarditis.

**Patient’s concerns:**

The patient is a 19-year-old young man who developed chest tightness and chest pain after catching a cold on April 14, 2021, which lasted for 2 days. The electrocardiogram showed sinus rhythm with ST-T change. The high-sensitivity cardiac troponin test result was 0.06 ng/mL, and the cardiac color ultrasound examination found no obvious structural and functional abnormalities.

**Diagnoses:**

viral myocarditis; fascicular ventricular tachycardia.

**Interventions:**

After 10 days of myocardial nutritional support treatment, the high-sensitivity cardiac troponin T test result was 0.004 ng/mL. Long-term follow-up was conducted after discharge, and as of December 2021, the reexamination indicators showed normal. However, in March 2023, the patient went to the hospital again due to palpitation and underwent an electrocardiogram. The electrocardiogram waveform showed wide Q wave-R wave-S wave complex ventricular tachycardia and complete right bundle branch block with left posterior fascicular block, and was diagnosed as fascicular ventricular tachycardia. Tachycardia, after cardiac electrophysiological examination and radiofrequency ablation treatment.

**Outcomes:**

The patient was no recurrence occurred during the 1 year follow-up, and the 24-hour ambulatory electrocardiogram showed sinus rhythm.

**Lessons:**

In clinical work, arrhythmia is common in patients with viral myocarditis, but cases of idiopathic branched ventricular tachycardia are rare, which may lead to a relative lack of understanding of the disease and is easily associated with supraventricular tachycardia with intraventricular differential conduction. Confusion, clinically is necessary to collect more relevant cases to analyze and explore its related mechanisms and treatments. At the same time, after treatment for children and adolescents with myocarditis, follow-up electrocardiograms can be used to avoid the occurrence of malignant arrhythmias.

## 1. Introduction

Fascicular ventricular tachycardia, also known as left ventricular idiopathic ventricular tachycardia, is a large reentrant ventricular tachycardia involving part of the Purkinje fiber system (mainly the left posterior branch) and ventricular myocardium. It is common in patients without in young patients with structural heart disease, the main symptom is palpitations, or they may not have any symptoms or signs. Fascicular ventricular tachycardia is divided into left anterior fascicular ventricular tachycardia, superior septal branch ventricular tachycardia, and left posterior fascicular ventricular tachycardia (LPF-VT), among which LPF-VT is more common. LPF-VT is characterized by right bundle branch block, left anterior fascicular block, and upward limb lead axis (left deviation or no man’s land) on the electrocardiogram. Regarding left posterior branch ventricular tachycardia, some researchers believe that LPF-VT large reentry originates from the ventricular myocardium, part of the LPF, slow conduction area, and special conductive P1 fibers.^[1]^ Although it is believed that this disease is mostly caused by a large reentry mechanism, the completeness of the specific reentry loop and the reentry site have not been reported. Some studies believe that reentrant loops are caused by abnormal conduction function of Purkinje fibers and can originate from any part of Purkinje fibers. Many studies have confirmed that LPF-VT is the most common form of idiopathic ventricular tachycardia in the left ventricle, and its reentry mechanism may be related to the reentry mechanism of slowly conducting verapamil-sensitive fibers and posterior bundles. One study also demonstrated verapamil-sensitive idiopathic left ventricular tachycardia with right bundle branch block architecture and left axis deviation suggestive of origin from the left posterior bundle branch. According to this mechanism, verapamil is the first choice for drug treatment, while catheter ablation is the only radical cure.^[2,3]^ Many scholars believe that Purkinje fibers with abnormal conduction function in LPF-VT show decreased conduction and verapamil sensitivity. Therefore, intravenous application of verapamil can effectively terminate this ventricular tachycardia episode and cannot control it. For patients with esophageal pacing therapy, the effect is ideal, but a complete cure requires catheter radiofrequency ablation.

## 2. Case presentation

**Figure 1.**
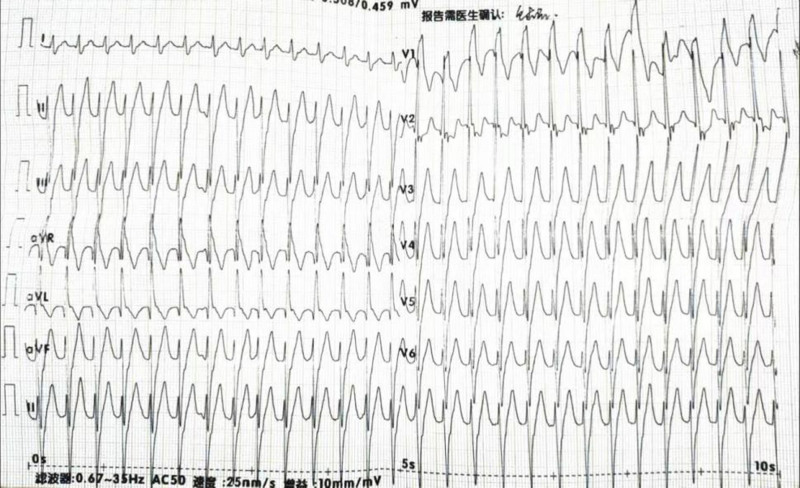
The electrocardiogram showed monomorphic right bundle branch block tachycardia with a QRS duration of 126 ms and right axis deviation. This manifestation was branched ventricular tachycardia. (Paper feeding speed is 25mm/s). QRS = Q wave-R wave-S wave.

The patient, a 19-year-old male, developed chest tightness and pain 2 days after catching a cold. There were no palpitations, fatigue, shortness of breath, or other discomforts. The symptoms were relieved after rest. He was admitted to the hospital on April 14, 2021. All physical signs were negative when he was admitted. Test: Lymphocyte percentage 18.3% (reference interval 20%–50%), white blood cells 8.53*109/L (reference value 3.5–9.5*109/L), neutrophil percentage 74.4%, high-sensitivity troponin T0.06 ng/mL, CK -MB1.05 ng/mL, myoglobin <21 ng/mL, C-reactive protein 2.19 mg/L; initial 12-lead electrocardiogram showed sinus bradycardia and ST-T changes, and no abnormality was found on cardiac color ultrasound; Consider viral myocarditis, rest, and nourish myocardial therapy. A 24-hour dynamic electrocardiogram performed on April 15, 2021, showed: 1. Sinus rhythm, second-degree type I atrioventricular block, the total number of heartbeats 85,833, slowest heart rate 42 beats/minute, the fastest heart rate was 106 beats/minute, and there were 2 pauses longer than 2 seconds, with the longest being 2.48 seconds. Considering that the patient has a high possibility of atrioventricular conduction due to myocarditis, he was given vitamin C to nourish the myocardium. The high-sensitivity troponin T0.015 ng/mL was rechecked on April 17, 2021; the 24 hour dynamic electrocardiogram was rechecked on April 20, 2021. The following prompts: 1. Sinus rhythm, There was no atrioventricular block, the slowest heart rate was 36 beats/min, the fastest heart rate was 112 beats/min, and there was 1 pause of >2 seconds, with the longest being 2.1 seconds; On April 23, 2021, the high-sensitivity troponin T0.004 ng/mL (reference value 0–0.014 ng/mL) was reviewed. The patient was discharged without any discomfort. He took a leave of absence from school for half a year after discharge. The electrocardiogram and cardiac enzyme indicators were regularly reviewed every 3 months. The patient suddenly suffered from palpitations on March 26, 2023, which lasted for about 4 hours without improvement. An electrocardiogram showed that the ventricular rhythm was regular and regular, with a Q wave-R wave-S wave duration of 126 ms and a frequency of 187 beats/min. It was considered to be branch-type ventricular tachycardia. (Fig. 1).

**Figure 2.**
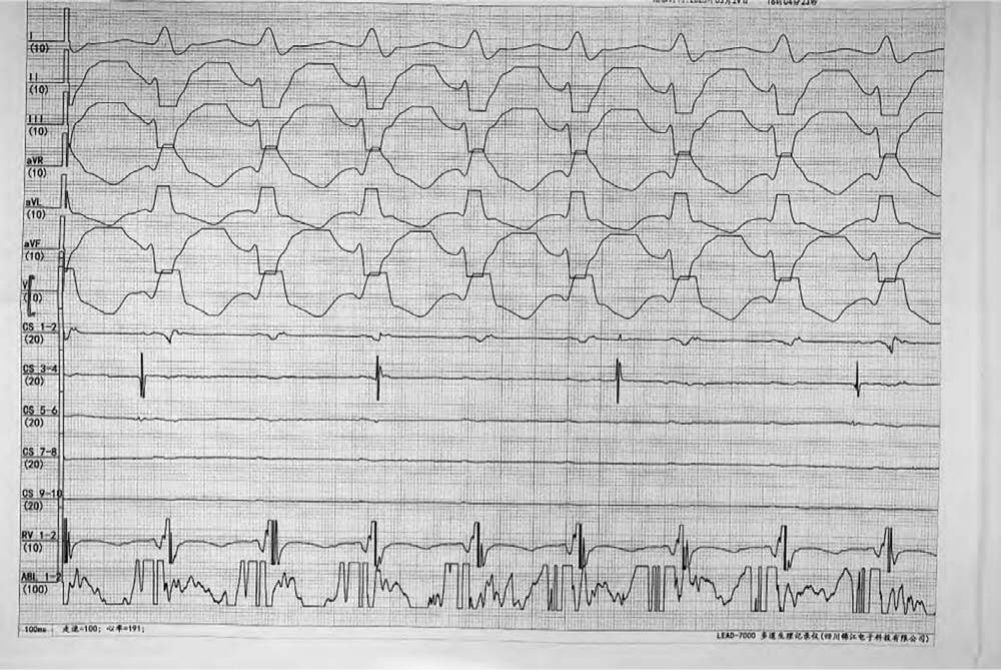
Intracavitary electrophysiology diagram during tachycardia, showing ventricular tachycardia (left posterior branch type).

**Figure 3.**
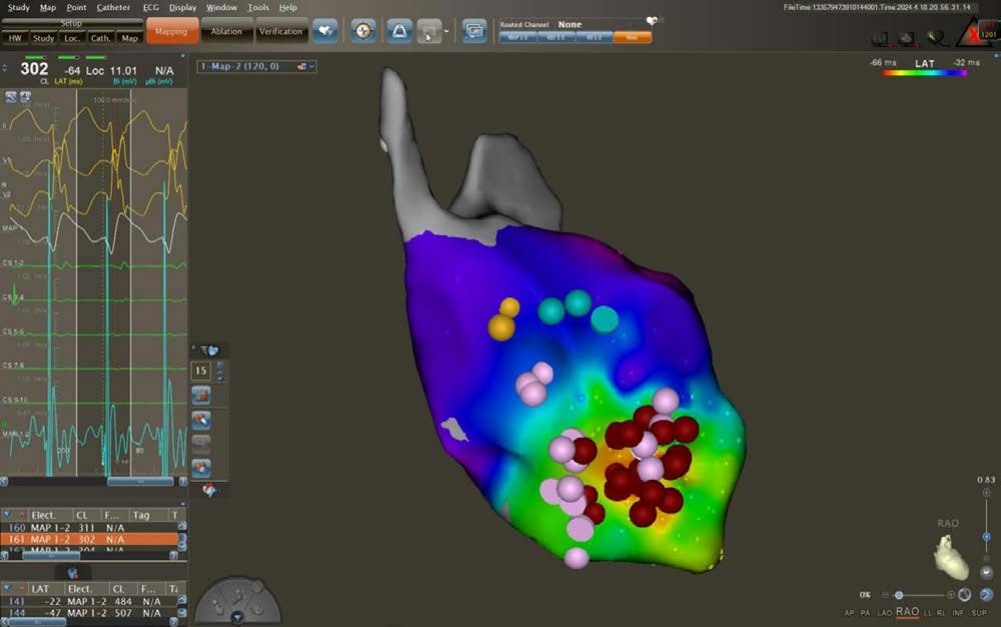
Picture during catheter operation (three-dimensional mapping and ablation picture). The pink point is the P point in the left posterior branch area, the blue point is the left anterior branch area, the yellow point is the His bundle, and the red point is the ablation point.

**Figure 4.**
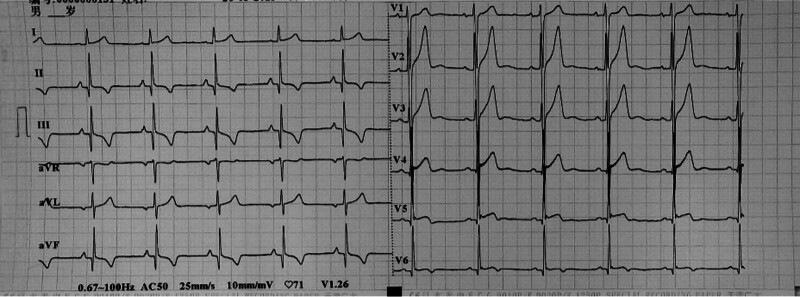
12-lead ECG after ablation.

**Figure 5.**
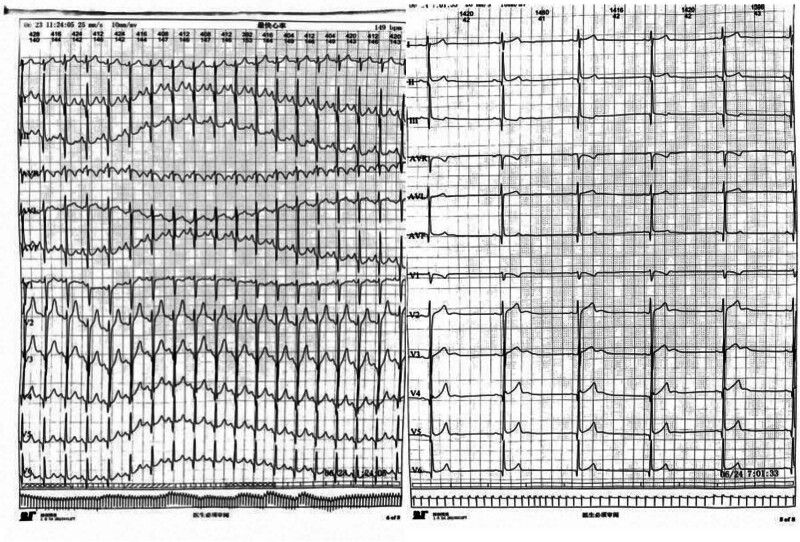
The 24-hour dynamic electrocardiogram reviewed 3 months after the operation showed sinus rhythm, with the fastest heart rate reaching 149 bpm and the slowest heart rate 42 bpm, with no pause longer than 2 seconds.

After being admitted to the hospital, chest CT, cardiac ultrasound, liver and kidney function, electrolytes, myocardial damage indicators, and ProBNP tests were performed, and the results were all normal. After signing the surgical consent form, an intracardiac electrophysiological examination was performed, and the results showed (Fig. 2): paroxysmal ventricular tachycardia (left posterior branch type). A cannula was inserted from the right femoral artery. After the ablation electrode was inserted, ventricular tachycardia activation mapping was performed in the left ventricle under 3-dimensional Carto mapping (Fig. 3). An advanced P potential was mapped in the left posterior branch area. After local ablation, the ventricular tachycardia was measured. The ablation was terminated quickly, and the ablation was consolidated for 120 seconds and observed for 20 minutes. There was no recurrence of electrical stimulation or atrioventricular jump. After the operation, the patient no longer felt palpitations. A review of the 12-lead electrocardiogram on the body surface showed that sinus rhythm was restored (Fig. 4). No recurrence occurred during the 1 year follow-up, and the review of the 24-hour dynamic electrocardiogram showed that sinus rhythm was restored (Fig. 5).

## 3. Discussion

Myocarditis is an inflammatory heart disease, mainly caused by viruses, but can also be induced by other infectious agents, toxic substances, drugs, systemic immune-mediated diseases, etc. Common viruses associated with viral myocarditis include adenovirus and enterovirus. Enteroviruses such as coxsackie group A virus, coxsackie group B virus, and echovirus. The exact mechanism of virus-related heart disease is still unclear. Enteroviral myocarditis occurs primarily in male adolescents and adults. Studies have mentioned that viruses infect cardiomyocytes by binding to common transmembrane receptors, thereby inducing direct myocardial damage,^[4]^ including cytoskeleton disruption, which can be triggered by inducing viral replication within host cells and subsequently lysing the cells to release the virus. Myocarditis. Regarding the occurrence of arrhythmias after myocarditis, some studies believe that first-degree atrioventricular block is a common and nonspecific finding in patients with acute myocarditis, occurring in >10% of patients with acute myocarditis.^[5]^ There are a large number of reports on ventricular arrhythmias in patients with myocarditis, and it is pointed out that the possibility of myocarditis should be considered for complex ventricular arrhythmias of unknown origin in children and adolescents.^[6]^ A study on active myocarditis concluded that the prevalence of nonsustained ventricular tachycardia and sustained ventricular tachycardia were 28% and 7%, respectively.^[7]^Arrhythmias caused by myocarditis may dominate the scar mechanism after myocardial injury. Many common ECG patterns are right bundle branch block, which are considered monomorphic and regular ventricular cardiac movements. Rapid speed is common in previous myocarditis^[8]^; and a comparison of the recurrence of ventricular tachycardia by 12 months after ventricular tachycardia ablation treatment was found to be more common in the former. The latter has high recurrence.^[9]^ A meta-analysis based on the nonrandom data that catheter ablation is an effective and long-lasting long-term treatment strategy for concurrent treatment by including data from 186 ventricular tachycardia after myocarditis. Patients with post-ventricular tachycardia with a lower incidence of symptoms, but based on the current study, a larger randomized controlled trial is required for long-term follow-up.^[10]^

According to the 2024 ACC expert consensus decision-making approach on the diagnosis and management strategies and standards of myocarditis: Diagnostic basis for the report of the American Society of Cardiology Solutions Oversight Committee, although the patient did not undergo a cardiomyopathological biopsy, the patient met the typical chest pain manifestations of myocarditis. There was a period of atrioventricular block, circulating cTn elevated, and a history of cold catching, so viral myocarditis was considered in this case.^[11]^ Arrhythmia occurred during hospitalization. We considered that it was caused by viral myocarditis. We were discharged from the hospital after rest and protection of myocardial treatment after the troponin and electrocardiogram were normal. After discharge, the electrocardiogram and test indicators were normal every 3 months after discharge. In March 2023, the patients electrocardiogram examination showed branch-type ventricular speed due to palpitations. Although the patient is a young male with normal heart structure and function, viral myocarditis itself may cause various arrhythmias. The 12-lead electrocardiogram in this patient showed monomorphic right bundle branch block tachycardia. The electrophysiology of the central organ is confirmed to be paroxysmal ventricular tachycardia (left posterior branch type). The trigger point of central arrhythmia during radiofrequency ablation is located at the apex, and it is believed that its site is an atypical trigger site of branched ventricular velocity. The treatment of cardiac radiofrequency ablation has achieved the goal of curing. Therefore, we consider the possibility that the patients arrhythmia is associated with viral myocarditis.

Studies have shown that branched ventricular tachycardia has a certain recurrence rate after radiofrequency ablation. There is currently no unified report on the recurrence rate. However, through this case, we learned that branched ventricular tachycardia after adolescent myocarditis can be cured by radiofrequency ablation. It may be related to his age, normal heart structure, and no other diseases. The disadvantage is that the follow-up time is short and it cannot be sure that he will not relapse in his life. In clinical work, arrhythmia is common in patients with viral myocarditis, but cases of idiopathic branched ventricular tachycardia are rare, which may lead to a relative lack of understanding of the disease and is easily associated with supraventricular tachycardia with intraventricular differential conduction. Confusion, clinically is necessary to collect more relevant cases to analyze and explore its related mechanisms and treatments. At the same time, after treatment for children and adolescents with myocarditis, follow-up electrocardiograms can be used to avoid the occurrence of malignant arrhythmias.

## Author contributions

**Data curation:** Wei Liu, Yun Chen.

**Investigation:** Wei Liu, Yu Bing Wang, Jie Feng, Yun Chen.

**Methodology:** Wei Liu, Jie Feng, Yun Chen.

**Writing – original draft:** Wei Liu.

**Writing – review & editing:** Wei Liu, Yu Bing Wang, Yun Chen.
